# Supplementary material for: A Critical View of the Application of the APEX Software (Aqueous Photochemistry of Environmentally-Occurring Xenobiotics) to Predict Photoreaction Kinetics in Surface Freshwaters
Source: Molecules. 2019 Dec 18;25(1):9. doi: 10.3390/molecules25010009 (PMC7017383; doi:10.3390/molecules25010009)
Supplement: Supplementary file 1 [file molecules-25-00009-s001.zip › 3.2.4_gcc-4.4.0.zip!/3.2.4_gcc-4.4.0/3.2.4_gcc-4.4.0/doc/octave/html/interpreter/Sending-Patches.html]

Sending Patches - Untitled


Next: Service,
Previous: Bug Reporting,
Up: Trouble


---

### E.6 Sending Patches for Octave

If you would like to write bug fixes or improvements for Octave, that is
very helpful. When you send your changes, please follow these
guidelines to avoid causing extra work for us in studying the patches.

If you don't follow these guidelines, your information might still be
useful, but using it will take extra work. Maintaining Octave is a lot
of work in the best of circumstances, and we can't keep up unless you do
your best to help.

- Send an explanation with your changes of what problem they fix or what
  improvement they bring about. For a bug fix, just include a copy of the
  bug report, and explain why the change fixes the bug.- Always include a proper bug report for the problem you think you have
    fixed. We need to convince ourselves that the change is right before
    installing it. Even if it is right, we might have trouble judging it if
    we don't have a way to reproduce the problem.- Include all the comments that are appropriate to help people reading the
      source in the future understand why this change was needed.- Don't mix together changes made for different reasons.
        Send them *individually*.

        If you make two changes for separate reasons, then we might not want to
        install them both. We might want to install just one.- Use diff -c to make your diffs. Diffs without context are hard
          for us to install reliably. More than that, they make it hard for us to
          study the diffs to decide whether we want to install them. Unidiff
          format is better than contextless diffs, but not as easy to read as
          -c format.

          If you have GNU diff, use diff -cp, which shows the name of the
          function that each change occurs in.- Write the change log entries for your changes.

            Read the ChangeLog file to see what sorts of information to put
            in, and to learn the style that we use. The purpose of the change log
            is to show people where to find what was changed. So you need to be
            specific about what functions you changed; in large functions, it's
            often helpful to indicate where within the function the change was made.

            On the other hand, once you have shown people where to find the change,
            you need not explain its purpose. Thus, if you add a new function, all
            you need to say about it is that it is new. If you feel that the
            purpose needs explaining, it probably does—but the explanation will be
            much more useful if you put it in comments in the code.

            If you would like your name to appear in the header line for who made
            the change, send us the header line.
